# Evaluation of cardiac strain parameters in pregnant women with and without iron deficiency anemia: a prospective case control study

**DOI:** 10.1186/s12884-025-08248-x

**Published:** 2025-10-24

**Authors:** Yunus Emre Özbebek, Eda Özden Tokalıoğlu, Ülkü Gürbüz Özbebek, Mehmet Erdoğan, Fatma Doğa Öcal, Dilek Şahin, Mehmet Akif Erdöl

**Affiliations:** 1 Department of Cardiology, Ankara Etlik City Hospital, Ankara, Turkey; 2 Department of Perinatology, Ankara Bilkent City Hospital, Ankara, Turkey; 3 Department of Obstetrics and Gynocology, Ankara Bilkent City Hospital, Ankara, Turkey; 4 Department of Cardiology, Ankara Bilkent City Hospital, Ankara, Turkey

**Keywords:** Pregnancy, Iron deficiency anemia, Echocardiography, Myocardial strain, Global longitudinal strain, Left ventricular function, Right ventricular function

## Abstract

**Background:**

Iron deficiency anemia (IDA) is highly prevalent in pregnancy and may impair cardiovascular function. Strain echocardiography can detect subclinical myocardial dysfunction, yet data on strain alterations in pregnant women with IDA are limited.

**Methods:**

This prospective comparative study included 80 pregnant women in the second or third trimester: 40 with IDA and 40 without anemia. All participants underwent comprehensive echocardiographic evaluation, including left ventricular global longitudinal strain (LV GLS) and right ventricular strain parameters. Laboratory iron indices were also assessed.

**Results:**

LV GLS was significantly reduced in the IDA group compared with controls (− 17.46 ± 2.14% vs. −19.34 ± 2.74%, *p* = 0.001). Basal right ventricular free-wall strain was more negative in the IDA group (*p* = 0.032), and systolic pulmonary artery pressure was higher (*p* = 0.005). ROC analysis identified an LV GLS cut-off of − 20.55% for detecting subclinical cardiac dysfunction (AUC = 0.699, sensitivity = 90.0%, specificity = 70.0%). Iron indices showed significant correlations with multiple strain parameters.

**Conclusions:**

IDA in pregnancy is associated with subclinical alterations in left and right ventricular strain despite preserved ejection fraction. Strain echocardiography may be a valuable tool for early detection of cardiac involvement in anemic pregnant women.

## Introduction

Iron deficiency anemia (IDA) is the most common nutritional deficiency worldwide and affects a substantial proportion of pregnant women, particularly in developing countries [1]. The increased iron demands of pregnancy, hemodilution, and insufficient dietary intake contribute to the high prevalence of IDA during gestation [2]. According to the World Health Organization, approximately 40% of pregnant women are anemic, with iron deficiency accounting for the majority of cases [3].

Beyond its well-known hematologic effects, iron deficiency has been increasingly recognized for its potential impact on cardiovascular function [4]. In non-pregnant populations, IDA has been linked to left ventricular remodeling, reduced exercise tolerance, and even adverse cardiovascular outcomes [5, 6]. During pregnancy, the cardiovascular system undergoes significant adaptive changes, including increased cardiac output and plasma volume expansion [7]. In the presence of anemia, these adaptations may impose additional hemodynamic stress on the maternal heart, potentially leading to subclinical myocardial dysfunction [8].

Strain echocardiography is a sensitive imaging modality capable of detecting early myocardial deformation before conventional measures such as ejection fraction are affected [9]. Global longitudinal strain (GLS) of the left and right ventricles offers valuable insights into subclinical systolic dysfunction and has been increasingly used in cardiology research [10]. However, to date, few studies have examined the relationship between iron deficiency anemia and cardiac strain parameters in pregnant women.

The present study aims to fill this gap by prospectively evaluating and comparing echocardiographic strain parameters in pregnant women with and without iron deficiency anemia. We hypothesize that IDA may be associated with impaired myocardial strain values, reflecting early cardiovascular involvement in this vulnerable population.

## Materials and methods

### Study design and population

This prospective, single-center observational study was conducted between January and June 2024 in the Departments of Obstetrics and Gynecology and Cardiology of our institution. A total of 80 pregnant women aged between 18 and 40 years with singleton pregnancies in their second or third trimesters were included. The participants were divided into two groups: 40 women diagnosed with iron deficiency anemia (IDA group) and 40 without anemia (control group).

Iron deficiency anemia was defined in accordance with the World Health Organization criteria for hemoglobin concentrations — <11.0 g/dL in the first and third trimesters or < 10.5 g/dL in the second trimester — in conjunction with the ferritin threshold of < 30 ng/mL as recommended by recent guidelines [11, 12].

Exclusion criteria included the presence of moderate to severe valvular heart disease, structural congenital heart anomalies, chronic renal or hepatic disease, multiple pregnancy, preeclampsia, preexisting diabetes mellitus or hypertension, any known hematologic or systemic inflammatory condition, and other causes of anemia such as vitamin B12 deficiency, folate deficiency, thalassemia, or anemia of chronic disease.

### Ethical approval

The study protocol was approved by the Institutional Ethics Committee of Ankara Bilkent City Hospital (Approval No: [E1-20-1143], Date: [28.10.2020]). Written informed consent was obtained from all participants prior to enrollment. The study was conducted in accordance with the ethical principles outlined in the Declaration of Helsinki.

### Laboratory parameters

Venous blood samples were collected from all participants for evaluation of the following laboratory parameters: hemoglobin, hematocrit, serum ferritin, serum iron, total iron binding capacity (TIBC), transferrin, white blood cell (WBC) count, neutrophils, lymphocytes, monocytes, red cell distribution width (RDW), platelet count, mean platelet volume (MPV), glucose, urea, creatinine, albumin, total protein, aspartate aminotransferase (AST), alanine aminotransferase (ALT), sodium, potassium, and thyroid-stimulating hormone (TSH).

### Echocardiographic assessment

All participants underwent transthoracic echocardiographic evaluation using a Philips EPIQ 7 ultrasound system equipped with a 3.5–5 MHz transducer. Examinations were performed by a single experienced cardiologist(Y.E.O) blinded to the patients’ anemia status. Standard two-dimensional and Doppler echocardiographic parameters were measured, including.

Left heart parameters: Ejection fraction (EF), left ventricular end-diastolic and end-systolic diameters (LVEDD, LVESD), left atrial diameter (LAEDD), septal and lateral tissue Doppler early diastolic velocities (Septal E, Lateral E), mitral inflow E and A waves (MiE, MiA), and global longitudinal strain of the left ventricle (LVGLS).

Right heart parameters: Tricuspid annular plane systolic excursion (TAPSE), systolic pulmonary artery pressure (SPAP), right ventricular free wall strain at basal, mid, and apical segments (RVFWSB, RVFWSM, RVFWSA), and right ventricular global longitudinal strain (RVGLS).

Speckle-tracking strain analysis was performed using the Philips QLab software based on apical four-chamber and long-axis views, in accordance with the current joint recommendations of the American Society of Echocardiography (ASE)and the European Association of Cardiovascular Imaging (EACVI) for chamber quantification and standardization of deformation imaging[13, 14]. 

### Statistical analysis

Statistical analyses were performed using IBM SPSS Statistics version 29.0 (IBM Corp.,Armonk, NY, USA). The Kolmogorov–Smirnov test was used to assess the normality of data distribution. Continuous variables were expressed as mean ± standard deviation (SD) for normally distributed data or median with interquartile range (IQR) for non-normally distributed data, while categorical variables were presented as frequencies and percentages.

Comparisons between the two groups were performed using the independent samples t-test for normally distributed continuous variables or the Mann–Whitney U test for non-normally distributed variables. The chi-square test or Fisher’s exact test was applied for categorical variables. Effect sizes were calculated using Cohen’s d for normally distributed variables and r for non-normally distributed variables.

Correlations between laboratory and echocardiographic parameters were assessed using Pearson or Spearman correlation coefficients, as appropriate. Receiver operating characteristic (ROC) curve analysis was conducted to evaluate the diagnostic performance of echocardiographic parameters for anemia, with the area under the curve (AUC), optimal cut-off values, sensitivity, and specificity determined using Youden’s index.

Multiple linear regression analyses were performed to identify independent predictors of myocardial strain parameters in relation to iron metabolism markers. A two-tailed p-value<0.05 was considered statistically significant. Multiple linear regression models included serum iron, total iron-binding capacity (TIBC), and transferrin entered a priori as independent variables. To avoid overfitting in this 80-participant cohort and given collinearity among hematologic indices, no additional covariates were included. Model assumptions were verified; collinearity was acceptable (all VIF < 1.5).

## Results

### Baseline characteristics

A total of 80 pregnant women (mean age: 28.88 ± 4.17 years) were included, with 40 in the iron deficiency anemia (IDA) group and 40 in the non-anemic control group. Baseline demographic, clinical, and laboratory data as shown in Table 1. There were no significant differences between groups in age, gestational age, or number of living children. However, gravidity was significantly higher in the IDA group (p = 0.019). As expected, hemoglobin, hematocrit, ferritin, and serum iron levels were significantly lower in the IDA group, whereas total iron-binding capacity (TIBC) and transferrin levels were significantly higher (all p < 0.001). The prevalence of hypertension was also higher in the IDA group (22.5% vs. 5.0%, p = 0.023).

**Table 1:** Baseline demographic, clinical, and laboratory characteristics

Parameter	Anemic (*n* = 40)	Non-anemic (*n* = 40)	*p*-value
Age (years)	29.15 ± 4.93	28.60 ± 3.29	0.559
Gestational age (weeks)	30.86 ± 4.50	29.49 ± 4.27	0.167
Gravida (number)	2.0 [2.75]	2.0 [1.00]	0.019
Living children (number)	1.0 [2.00]	1.0 [1.00]	0.058
Hemoglobin (g/dL)	10.42 ± 1.40	12.95 ± 0.92	< 0.001
Hematocrit (%)	32.69 ± 4.02	39.58 ± 2.81	< 0.001
Ferritin (ng/mL)	7.15 [10.30]	48.6 [27.00]	< 0.001
Iron (µg/dL)	39.5 [18.25]	97.0 [54.50]	< 0.001
TIBC (µg/dL)	468.88 ± 84.96	339.70 ± 84.62	< 0.001
Transferrin (mg/dL)	385.5 [49.50]	255.0 [71.75]	< 0.001
Diabetes mellitus	5 (12.5%)	2 (5.0%)	0.235
Hypertension	9 (22.5%)	2 (5.0%)	0.023

Values are expressed as mean ± standard deviation (SD) for normally distributed variables, median [interquartile range] for non-normally distributed variables, and number (percentage) for categorical variables. p-values were calculated using the independent-samples t-test, Mann–Whitney U test, or Chi-square/Fisher’s exact test, as appropriate

### Echocardiographic Findings

Echocardiographic parameters according to anemia status are shown in Table 2. Left ventricular global longitudinal strain (LV GLS) was significantly reduced in the IDA group compared to controls (−17.46 ± 2.14% vs. −19.34 ± 2.74%, p = 0.001; large effect size, d = −0.763). Additionally, ejection fraction (EF) was significantly higher in the IDA group (65.0 [IQR 5.0] vs. 60.0 [IQR 5.0], p = 0.011). Right ventricular free wall strain at the basal segment (RV FW Sb) was more negative in the IDA group (−27.55 [14.13] vs. −22.70 [6.98], p = 0.032). Systolic pulmonary artery pressure (SPAP) was also significantly higher in the IDA group (25.0 [8.0] vs. 20.0 [5.0] mmHg, p = 0.005). No significant differences were observed in LVEDD, LVESD, TAPSE, or other right ventricular strain segments.

**Table 2:** Echocardiographic parameters by anemia status

Parameter	No Anemia	Anemia	*p*-value	Effect size
EF (%)	60.0 [5.0]	65.0 [5.0]	0.011	−0.283
LVEDD (cm)	4.70 ± 0.27	4.75 ± 0.27	0.365	−0.204
LVESD (cm)	3.05 [0.50]	3.15 [0.30]	0.200	−0.143
LV GLS (%)	−19.34 ± 2.74	−17.46 ± 2.14	0.001	−0.763
Septal e′ (cm/s)	10.0 [2.30]	9.8 [1.57]	0.355	−0.103
SPAb (%)	20.0 [5.0]	25.0 [8.0]	0.005	−0.315
RVS (%)	16.0 [2.28]	15.4 [2.82]	0.250	−0.129
RV FW Sb (%)	−22.70 [6.98]	−27.55 [14.13]	0.032	−0.239
RV FW Sm (%)	−22.65 [5.70]	−20.90 [10.15]	0.900	−0.014
RV FW Sa (%)	−20.91 ± 6.29	−20.93 ± 6.79	0.988	0.003
TAPSE (cm)	2.50 [0.30]	2.60 [0.40]	0.528	−0.071

Values are presented as mean ± SD for normally distributed variables and median [IQR] for non-normally distributed variables. Effect sizes are reported as Cohen’s d for normally distributed variables and r for non-normally distributed variables. Negative values in strain parameters indicate myocardial shortening

### Correlation analyses

Correlation results between strain parameters and laboratory variables are summarized in Table 3. LV GLS showed a significant negative correlation with serum iron (r = −0.266, p = 0.017) and positive correlations with TIBC (r= 0.294, p = 0.008) and transferrin (r = 0.286, p = 0.010). RV FW Sb demonstrated a significant positive correlation with serum iron (ρ = 0.317, p = 0.004) and a negative correlation with TIBC (ρ = −0.369, p < 0.001). SPAP correlated negatively with serum iron (ρ = −0.431, p < 0.001) and positively with TIBC (ρ = 0.374, p < 0.001) . A borderline negative correlation was also observed between hemoglobin and LV GLS (r = −0.210, p = 0.062).

**Table 3:** Correlation between echocardiographic strain parameters and laboratory variables

Strain parameter	Laboratory parameter	Correlation (*r*/ρ)	*p*-value
LV GLS (%)	Ferritin	r −0.112	0.322
	Hemoglobin	r −0.210	0.062
	Hematocrit	r −0.177	0.117
	Iron	**r −0.266**	**0.017**
	TIBC	**r 0.294**	**0.008**
	Transferrin	**r 0.286**	**0.01**
RV FW Sa (%)	Ferritin	r 0.046	0.685
	Hemoglobin	r −0.167	0.138
	Hematocrit	r −0.115	0.31
	Iron	r −0.043	0.707
	TIBC	r −0.101	0.374
	Transferrin	r −0.070	0.54
RV GLS (%)	Ferritin	r −0.118	0.299
	Hemoglobin	r −0.136	0.228
	Hematocrit	r −0.079	0.487
	Iron	r 0.079	0.484
	TIBC	**r −0.265**	**0.017**
	Transferrin	r −0.035	0.757
TAPSE (cm)	Ferritin	ρ −0.054	0.637
	Hemoglobin	ρ 0.087	0.441
	Hematocrit	ρ 0.081	0.473
	Iron	ρ −0.148	0.191
	TIBC	ρ 0.128	0.258
	Transferrin	ρ 0.010	0.927
RV FW Sm (%)	Ferritin	ρ −0.175	0.121
	Hemoglobin	ρ −0.079	0.484
	Hematocrit	ρ −0.009	0.935
	Iron	ρ −0.045	0.693
	TIBC	ρ −0.188	0.095
	Transferrin	ρ −0.002	0.986
RV FW Sb (%)	Ferritin	ρ 0.120	0.29
	Hemoglobin	ρ 0.156	0.167
	Hematocrit	ρ 0.139	0.218
	Iron	**ρ 0.317**	**0.004**
	TIBC	**ρ −0.369**	**< 0.001**
	Transferrin	ρ −0.098	0.389
RVS (%)	Ferritin	ρ 0.172	0.127
	Hemoglobin	ρ 0.091	0.422
	Hematocrit	ρ 0.057	0.613
	Iron	ρ 0.098	0.385
	TIBC	ρ 0.059	0.602
	Transferrin	ρ −0.059	0.602
SPAb (%)	Ferritin	**ρ −0.264**	**0.018**
	Hemoglobin	**ρ −0.240**	**0.032**
	Hematocrit	ρ −0.167	0.139
	Iron	**ρ −0.431**	**< 0.001**
	TIBC	**ρ 0.374**	**< 0.001**
	Transferrin	ρ 0.179	0.112

r = Pearson correlation coefficient, ρ = Spearman’s rank correlation coefficient

Values in bold indicate statistically significant correlations (*p* < 0.05)

### Multivariate regression analysis

Multiple linear regression analyses assessing the association between iron metabolism markers and strain parameters are shown in Table 4. No iron-related marker remained independently associated with LV GLS (iron: β =−0.110, p = 0.390; TIBC: β = 0.183, p = 0.137; transferrin: β = 0.165, p = 0.186; model F = 3.804, p = 0.013; R² = 0.131; adjusted R² = 0.096). In contrast, higher TIBC was independently associated with worse RV GLS (B =−0.013, 95% CI −0.025 to −0.002; β = −0.300; p = 0.019), more negative RV FW Sb (B = −0.023, 95% CI −0.042 to −0.004; β = −0.300; p = 0.016), and more negative RV FW Sm (B = −0.020, 95% CI −0.035 to −0.005; β = −0.332; p = 0.010).

**Table 4:** Multiple linear regression analysis of strain parameters in relation to iron metabolism markers

Dependent Variable	Predictor	B (Unstd.)	SE	β (Std.)	t	*p*-value	95% CI for B
LV GLS (%)	Iron (µg/dL)	−0.005	0.006	−0.110	−0.865	0.390	−0.017 to 0.007
	TIBC (µg/dL)	0.005	0.003	0.183	1.502	0.137	−0.002 to 0.011
	Transferrin (mg/dL)	0.005	0.004	0.165	1.334	0.186	−0.003 to 0.013
RV GLS (%)	Iron (µg/dL)	−0.002	0.012	−0.019	−0.141	0.888	−0.025 to 0.022
	TIBC (µg/dL)	−0.013	0.006	−0.300	−2.388	0.019	−0.025 to −0.002
	Transferrin (mg/dL)	0.004	0.007	0.071	0.558	0.578	−0.010 to 0.018
RV FW S′ (Apical, %)	Iron (µg/dL)	−0.017	0.017	−0.137	−1.021	0.311	−0.050 to 0.016
	TIBC (µg/dL)	−0.008	0.008	−0.128	−0.994	0.323	−0.024 to 0.008
	Transferrin (mg/dL)	−0.007	0.010	−0.084	−0.640	0.524	−0.027 to 0.014
RV FW S′ (Basal, %)	Iron (µg/dL)	0.024	0.020	0.158	1.239	0.219	−0.015 to 0.064
	TIBC (µg/dL)	−0.023	0.009	−0.300	−2.462	0.016	−0.042 to −0.004
	Transferrin (mg/dL)	0.009	0.012	0.090	0.727	0.470	−0.015 to 0.033
RV FW S′ (Mid, %)	Iron (µg/dL)	−0.022	0.016	−0.185	−1.426	0.158	−0.054 to 0.009
	TIBC (µg/dL)	−0.020	0.007	−0.332	−2.660	0.010	−0.035 to −0.005
	Transferrin (mg/dL)	−0.001	0.010	−0.007	−0.052	0.959	−0.020 to 0.019

LV GLS = Left ventricular global longitudinal strain; RV GLS = Right ventricular global longitudinal strain; RV FW S′ = Right ventricular free-wall systolic velocity by tissue Doppler imaging (Apical, Mid, Basal segments); TIBC = Total iron-binding capacity. Standardized (β) and unstandardized (B) regression coefficients are presented with their standard errors (SE) and 95% confidence intervals (CI). Statistically significant p-values (< 0.05) are shown in bold

### Receiver operating characteristic (ROC) analysis

**Fig. 1:**
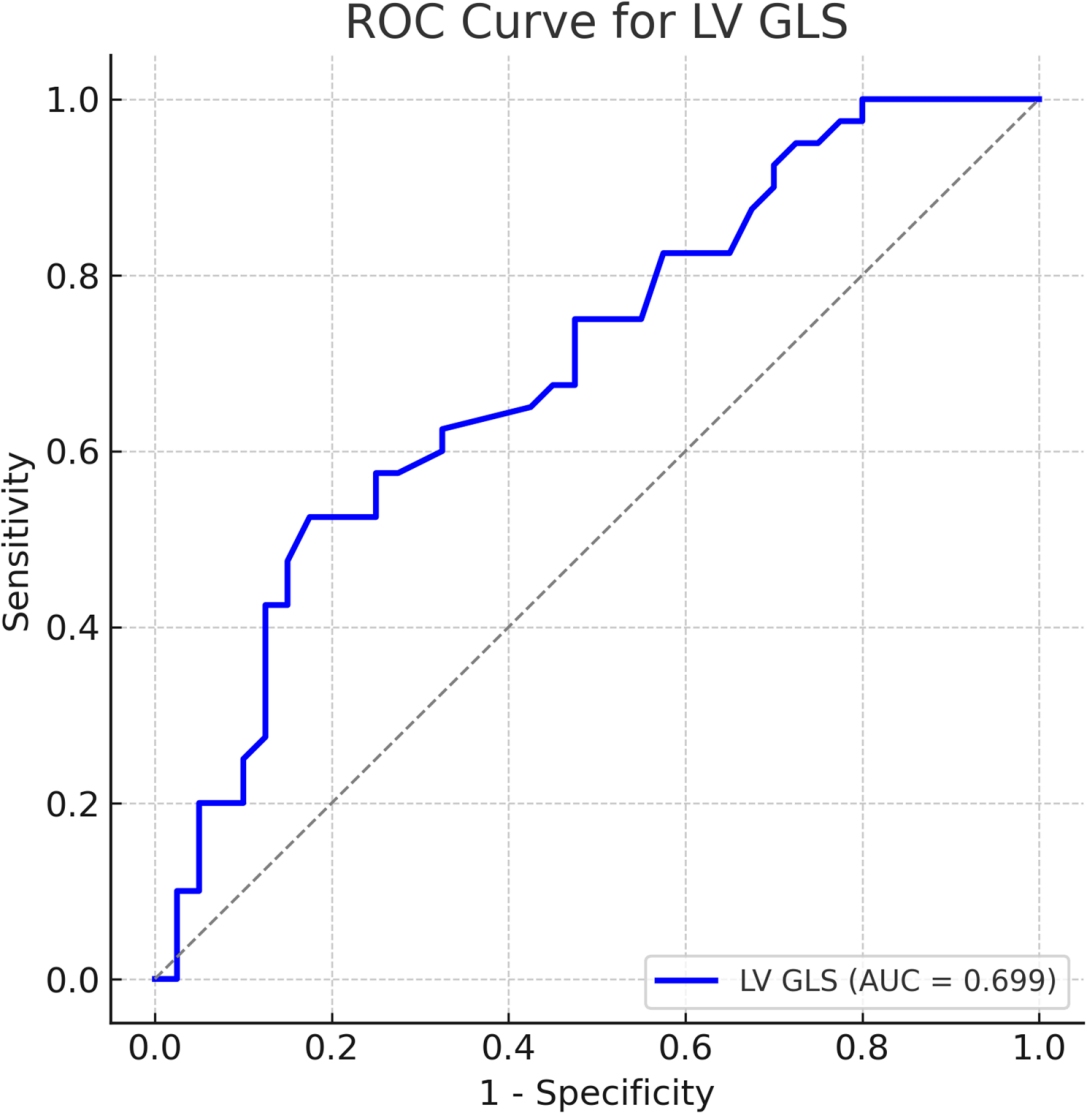
ROC analysis of LV GLS for detection of subclinical cardiac dysfunction Declarations Ethics approval and consent to participate The study protocol was approved by the Institutional Ethics Committee of Ankara Bilkent City Hospital (Approval No: E1-20-1143, Date: 28.10.2020). Written informed consent was obtained from all participants Consent for publication Not applicable Availability of data and materials The datasets used and/or analyzed during the current study are available from the corresponding author on reasonable request

ROC analysis of LV GLS for detecting subclinical cardiac dysfunctionis illustrated in Figure 1. LV GLS demonstrated a significant discriminative ability with an area under the curve (AUC) of 0.699 (95% CI: 0.580–0.819, p = 0.002). The optimal cut-off value determined by Youden’s index was −20.55%, yielding a sensitivity of 90.0% and a specificity of 70.0%.

## Discussion

This prospective study demonstrated that pregnant women with iron deficiency anemia (IDA) have significantly impaired left ventricular global longitudinal strain (LV GLS) compared with non-anemic controls. Additionally, systolic pulmonary artery pressure (SPAP) was higher and basal right ventricular free-wall strain values were more negative in the IDA group. These findings fill a gap in the literature, as evidence regarding maternal myocardial strain changes in IDA during pregnancy remains scarce. Interestingly, despite a slightly higher median ejection fraction in the IDA group, LV GLS values were significantly less negative, highlighting the superior sensitivity of strain imaging for detecting subtle myocardial dysfunction before changes in conventional measures become apparent.

According to the World Health Organization, iron deficiency is the most prevalent nutritional deficiency in pregnancy, with iron deficiency anemia affecting approximately 36.5% of pregnant women worldwide[15]. Despite its high prevalence, routine screening for iron deficiency during pregnancy is not universally implemented, and the United States Preventive Services Task Force has concluded that current evidence is insufficient to determine the balance of benefits and harms of such screening in pregnant individuals [16]. Moreover, diagnostic criteria for iron deficiency in pregnancy are inconsistent: hemoglobin concentration alone is frequently used, which detects anemia but may miss earlier functional impairment [2]. Ferritin remains the most reliable early marker, yet thresholds vary between recommendations (WHO <15 µg/L vs. recent guidelines suggesting <30 µg/L) [11, 17]. Alternative markers such as soluble transferrin receptor and total body iron can be useful, especially given that ferritin levels may be influenced by inflammation [18].

Previous research utilizing both three-dimensional speckle-tracking echocardiography (3DSTE) and left ventricular pressure–strain loop (LV-PSL) analysis in non-pregnant patients with IDA has demonstrated that when hemoglobin levels fall to 6–9 g/dL, marked left ventricular remodeling, significantly reduced GLS, and impaired myocardial work indices occur, highlighting these modalities as sensitive tools for detecting early subclinical systolic dysfunction[5, 19].. Cui et al. demonstrated that patients with iron deficiency anemia exhibited significantly reduced GLS, GWI, GCW, and GWE, alongside increased GWW, despite preserved ejection fraction, highlighting the utility of LV-PSL in detecting early subclinical LV systolic dysfunction with high sensitivity and specificity[5]. Mechanistically, disturbances in iron homeostasis can impair oxygen delivery and disrupt iron-dependent myocardial energetics, potentially contributing to ventricular dysfunction. These alterations are consistent with evidence from cardiovascular disease populations, where iron imbalance and related pathways have been implicated in myocardial injury and adverse remodeling[20–22].. The present study extends these observations to a pregnant cohort, underscoring LV GLS as a sensitive marker of early myocardial involvement.

Although LV GLS was significantly reduced in the IDA group, the discriminatory ability was moderate (AUC = 0.699). Therefore, LV GLS should be interpreted as a potentially useful adjunctive marker associated with IDA status, rather than a definitive diagnostic tool for subclinical dysfunction. SPAP was significantly higher in the IDA group; however, the absolute difference was small, and its clinical significance in otherwise healthy pregnant women is likely limited. Interestingly, basal RV free-wall strain values were more negative in the IDA group, which may represent a compensatory augmentation of basal RV contractility in response to increased pulmonary pressures or volume load, rather than intrinsic systolic dysfunction[23]. This study has limitations. Being a single-center study with a relatively modest sample size may limit the generalizability of the findings. Given that cardiac strain parameters can be influenced by physiological changes in pregnancy, distinguishing the direct effects of iron deficiency anemia from gestational cardiovascular adaptations remains challenging, as highlighted in previous studies[24, 25]. Another limitation is the lack of postpartum echocardiographic follow-up, which could have provided valuable insights into the reversibility of strain alterations after delivery. Future research should adhere to standardized deformation imaging protocols, as recommended by current ASE/EACVI guidelines[13, 14] to ensure reproducibility and facilitate inter-study comparisons. Longitudinal interventional studies assessing whether correction of IDA can normalize strain values and improve maternal–fetal outcomes are warranted.

Additionally, speckle-tracking echocardiography is subject to several technical limitations, including inter-vendor variability, dependence on frame rate settings, sensitivity to loading conditions, and potential influence of extrinsic mechanical factors such as a narrow anteroposterior thoracic diameter[26–28]. Moreover, reproducibility testing for strain measurements was not performed, which limits the assessment of measurement reliability.

## Conclusion

In pregnant women, iron deficiency anemia is associated with significant alterations in myocardial strain, particularly reduced left ventricular global longitudinal strain, despite preserved ejection fraction. LV GLS may serve as a potentially useful adjunctive marker for early myocardial involvement in this population, but its role in screening should be interpreted with caution and confirmed by future prospective studies.

## Data Availability

The datasets used and/or analyzed during the current study are available from the corresponding author on reasonable request.

## Abbreviations

LV GLS: Left ventricular global longitudinal strain
RV GLS: Right ventricular global longitudinal strain
RV FW S′: Right ventricular free-wall systolic velocity by tissue Doppler imaging (Apical, Mid, Basal segments)
TIBC: Total iron-binding capacity
EF: Ejection fraction
SPAP: Systolic pulmonary artery pressure
TAPSE: Tricuspid annular plane systolic excursion
r: Pearson correlation coefficient
ρ: Spearman’s rank correlation coefficient
